# Factors mediating performance monitoring in humans—from context to personality

**DOI:** 10.3389/fnhum.2013.00023

**Published:** 2013-01-31

**Authors:** Patrizia Thoma, Christian Bellebaum

**Affiliations:** Department of Neuropsychology, Institute of Cognitive Neuroscience, Ruhr-University BochumBochum, Germany

In everyday life, we constantly monitor our behavior and adapt our responses following performance errors and feedback information from the environment. Receiving social, monetary or some other type of feedback can encourage us to continue with a specific action or may lead us to discontinue the same behavior. Additionally, we daily observe other people performing various tasks and we can not only learn from their errors or the feedback they receive but also infer how they feel. Whether we feel sorry for their failures and happy about their successes may depend on our empathic concern and on the relationship to the observed person.

The present e-book, which is based on our Frontiers Research Topic entitled “Factors mediating performance monitoring in humans—from context to personality,” encompasses both reviews and original research articles which explore the neurocognitive mechanisms supporting performance monitoring providing a link to contextual factors or personality traits.

The overarching theoretical framework for the current Research Topic is presented in three review articles: Thoma and Bellebaum (2012) aimed to link the electrophysiological correlates of performance monitoring, in particular, the mediofrontal negative components error-related negativity (ERN) and feedback-related negativity (FRN), to the concept of empathy. One of the main conclusions they reached is that empathy might be more strongly related to observational than to active learning. This makes sense intuitively given that learning from another person's errors or performance feedback might also involve inferring how the other person feels in response to these events. van Noordt and Segalowitz (2012) adopted a broader perspective on this issue. They reviewed the performance monitoring literature taking into account a variety of potential interindividual differences (such as temperament, different genetic endowments, and various personality factors) as well as different task contexts and linked them to MPFC functioning, as reflected by the mediofrontal negativities. The authors emphasize the highly complex effects of these factors and their interactions on performance monitoring. Brown and Brüne (2012) surveyed the related social neuroscience literature from yet a different angle by focusing on the role of predictive internal representations of one's own and other people's actions, emotions, and outcomes for successful performance monitoring. They postulate that non-social predictive mechanisms, such as prediction error and efference copy signals, also contribute to the processing of social information.

Two original studies in our e-book highlight the importance of the personality dimension discussed in all three previous articles in relation to performance monitoring: Hoffmann et al. (2012) investigated the relationship between the ERN and personality factors, finding a negative association between the ERN and the personality dimensions of “Openness,” “Impulsiveness,” and “Emotionality” as well as a positive relationship between the ERN and “Social Orientation.” The authors conclude that the way people respond to their errors is modulated by their overall emotional and social rigidity. In a comment to this study, Tops and Koole (2012) extended the discussion of the findings arguing that traits related to higher task engagement predict ERN amplitude. Unger et al. (2012), on the other hand, reported a positive association between higher punishment sensitivity and higher FRN amplitudes, independent of feedback validity, which at the same time appeared to be related to poorer behavioral learning performance.

Three further original studies addressed the meaning of contextual factors for performance monitoring: Wu et al. (2011) investigated how recipients in the Ultimatum Game responded when they were not only informed about their own offers but also about the offers of other recipients. The results suggest that, on a neural level, evaluation of fairness in asset division involves an earlier automatic component (mediofrontal negativity) responding to fairness at an abstract level and a later appraisal process (late positive potential) affected by social comparison. Zhang et al. (2012) investigated neural responses to feedback stimuli with a social dimension (female faces). Participants were asked to judge the attractiveness of blurred faces and were shown unblurred faces as feedback. A late FRN-like component showed higher amplitudes in response to feedback faces that were inconsistent with the initial attractiveness judgment than to faces consistent with the judgment. For wave forms in the P300 time window, an opposite effect was found only with more sophisticated data analysis techniques involving a principle component analysis. The authors conclude that complex social feedback stimuli are processed in a similar way as non-social feedback stimuli. Schuermann et al. (2012) investigated how low and high risk for gains and losses affected event-related potentials. FRN amplitudes were enhanced following high-risk decisions but only for gains, while the early positivity (P200) was increased in response to losses following high-risk choices. Finally, P300 amplitudes were increased in high-risk decisions, and in an additive way, following losses compared to gains, suggesting that the P300 may process additional information related to the motivational significance of the processed rewards.

The authors of all three review articles (Brown and Brüne, 2012; Thoma and Bellebaum, 2012; van Noordt and Segalowitz, 2012) advocate the investigation of clinical populations to inform theories about the interactions between context, personality, and performance monitoring. Accordingly, three articles in this e-book involved subclinical or clinical populations: Pfabigan et al. (2011) demonstrated that in comparison with individuals scoring low on anti-social personality traits, individuals with more pronounced antisocial personality traits show enhanced FRN amplitudes to monetary, but not to social feedback. This highlights that these individuals might attribute higher motivation valence to financial assets. Morris et al. (2011) reported that while schizophrenia patients showed diminished ERN amplitudes relative to controls following erroneous responses, groups did not differ on feedback-related activity. Using fMRI, Mainz et al. (2012) investigated the effects of alcohol-related cue exposure on inhibition performance in alcohol-dependent participants. While they did not find any behavioral effects, exposition to alcohol cues was associated with subjectively stronger urges to drink and differential neural activation in amygdala and hippocampus.

Although healthy aging does of course not constitute a pathological condition, it is accompanied by a number of neurobehavioral changes which may alter performance monitoring. Drueke et al. (2012) investigated the effects of performance feedback on executive control, as exerted during a flanker task, in younger and older adults. They found that, although performance feedback improved executive performance in younger individuals, this was not the case in older adults. Error rates, on the other hand, were increased by performance feedback in both groups.

Taken together, we hope that the diverse articles comprised in our e-book may help to illustrate some of the complexities and exiting new developments regarding the intricate relationships between different environmental and personality factors affecting performance monitoring.
